# Sarcopenia index (serum creatinine/cystatin C ratio) independently identifies sarcopenia risk and mortality in older hospitalized patients with type 2 diabetes mellitus: a retrospective analysis

**DOI:** 10.3389/fendo.2026.1940459

**Published:** 2026-09-01

**Authors:** Tingting Lv, Xiaohui Feng, Jiehua Zhu, Tingjun Hu, Zehua Luo, Sheng Ge, Chenxi Ren

**Affiliations:** 1 Department of Clinical Nutrition, Shanghai Sixth People’s Hospital Affiliated to Shanghai Jiao Tong University School of Medicine, Shanghai, China; 2 Department of Clinical Nutrition, College of Health Science and Technology, Shanghai Jiao Tong University School of Medicine, Shanghai, China; 3 Department of Gerontology, Shanghai Sixth People’s Hospital, Shanghai Jiao Tong University School of Medicine, Shanghai, China

**Keywords:** diabetes mellitus, mortality, old, sarcopenia index, sarcopenia risk

## Abstract

Sarcopenia is increasingly recognized as a complication of type 2 diabetes mellitus (T2DM); however, simple screening tools for hospitalized older adults remain limited. The sarcopenia index (SI), calculated as serum creatinine (mg/dL)/cystatin C (mg/L) × 100, has been proposed as a readily available biomarker of muscle mass. However, its clinical utility and optimal cut-off value in older Chinese inpatients with T2DM are unclear. This retrospective study included 296 hospitalized older patients with T2DM from the Department of Geriatrics at Shanghai Sixth People’s Hospital between July 2019 and April 2020 and evaluated all-cause mortality. Sarcopenia risk was diagnosed according to the Asian Working Group for Sarcopenia 2019 consensus criteria. Logistic regression was performed to assess the association between SI and sarcopenia risk. Receiver operating characteristic (ROC) curve analysis was used to determine the optimal cut-off value, and Cox regression was used to evaluate the association between SI and mortality during follow-up (median 212 days). SI was significantly lower in patients with sarcopenia risk than in those without sarcopenia risk (68.0 ± 21.6 vs. 77.4 ± 20.0, *P* = 0.006). After adjusting for potential confounders, each 1-SD increase in SI was associated with a 68% lower risk of sarcopenia (adjusted OR = 0.32, 95% CI: 0.15–0.69). The optimal SI cutoff for detecting sarcopenia risk was 77.3 (AUC = 0.68, 95% CI: 0.59–0.77). Higher SI corresponded to a reduced risk of all-cause mortality during follow-up (median 212 days). after adjusting for potential confounders (HR per 1-SD = 0.70, 95% CI: 0.48–0.97). Subgroup analyses further revealed that the discriminative ability of SI varied by renal function, underscoring the necessity of kidney-specific validation. These findings suggest that SI is independently associated with sarcopenia risk and mortality in hospitalized older Chinese patients with T2DM and may provide a simple, low-cost tool for early sarcopenia screening in this high-risk population.

## 1 Introduction

Sarcopenia is a progressive skeletal muscle disorder characterized by loss of muscle mass, strength, and physical function, leading to adverse outcomes, including falls, functional decline, frailty, and mortality (1). Among older adults, the global prevalence of sarcopenia is approximately 10–27% (2) and is higher in those with chronic diseases. Patients with diabetes mellitus (DM) are particularly vulnerable to muscle loss, highlighting the intersection between sarcopenia and chronic disease. Recent meta-analyses have revealed that sarcopenia is two to three times more prevalent among patients with DM than among non-diabetic individuals (3, 4). Potential mechanisms include insulin resistance, chronic inflammation, and accumulation of advanced glycation end products, which accelerate protein degradation and impair muscle synthesis (5). Sarcopenia is therefore increasingly considered a novel complication of diabetes, particularly type 2 DM (T2DM).

Reference standards for muscle mass assessment include dual-energy X-ray absorptiometry (DEXA), computed tomography (CT), and magnetic resonance imaging (MRI). However, these techniques are limited in routine practice by high cost, access, radiation exposure in the case of CT, and technical complexity. Therefore, simple, low-cost, and readily available screening tools are needed.

The sarcopenia index (SI), defined as the ratio of serum creatinine (Cr) to cystatin C (CysC), has emerged as a promising alternative. Creatinine is predominantly derived from skeletal muscle, and its production remains relatively stable when muscle mass is constant (6). However, serum creatinine is also influenced by renal function through changes in excretion (7). Impaired excretion can increase creatinine independently of muscle mass, thereby confounding its interpretation as a muscle mass marker (8). In contrast, cystatin C is produced by all nucleated cells at a constant rate, is minimally affected by muscle mass or nutritional status, and reliably reflects glomerular filtration rate (9). The ratio of Cr to CysC theoretically minimizes the confounding effect of renal function and mainly reflects differences in skeletal muscle mass. Accordingly, a low SI suggests reduced muscle mass (10). SI has been validated in several populations, including critically ill patients and those with chronic kidney disease, as a correlate of measured muscle mass and a predictor of adverse clinical outcomes (11, 12).

Nevertheless, the clinical utility of the SI and its optimal cutoff for identifying sarcopenia in older Chinese patients with T2DM remain unclear. Therefore, this study aims to evaluate the clinical utility of the SI and determine its optimal cutoff for identifying sarcopenia risk in this population.

## 2 Methods

### 2.1 Study population

This retrospective study consecutively enrolled 425 diabetic patients with diabetes who were hospitalized in the Department of Geriatrics at Shanghai Jiao tong University Affiliated Sixth People’s Hospital between July 2019 and April 2020. The inclusion criteria were as follows (1): age ≥ 65 years; (2) availability of complete nutritional risk and sarcopenia assessments; and (3) a diagnosis of T2DM. The exclusion criteria were as follows: patients aged <65 years (n=2), those undergoing hemodialysis (n=3), peritoneal dialysis (n=1), patients with acute renal insufficiency (n=3), patients with a history of malignant tumor or cachexia (n=15), and patients in critical condition (n=69), including those with acute myocardial infarction, acute heart failure, acute stroke, or severe infection; patients unable to communicate (n=19); and bedridden patients (n=17) with edema. After these exclusions, 296 patients were included in the final analysis (**Supplementary Figure 1**). This study was approved by the Ethics Committee of Shanghai Jiaotong University Affiliated Sixth People’s Hospital [2026-KY-281(K)]. All participants provided written informed consent in accordance with the principles of the Declaration of Helsinki.

### 2.2 Data collection

Basic patients’ detail about medical history and lifestyle, including smoking, drinking status and age, were confirmed through examination of medical records by experienced physicians. Overweight and central obesity were defined according to the 2001 National Cholesterol Education Program Adult Treatment Panel III (NCEP ATPIII) working definition (13). Waist circumference thresholds followed Asian population criteria. Overweight was defined as a body mass index (BMI) ≥ 25 kg/m², and central obesity was defined as a waist circumference > 90 cm in men and > 80 cm in women. The Modification of Diet in Renal Disease (MDRD) equation was used to calculate estimated glomerular filtration (eGFR). Participants were classified as having renal dysfunction if eGFR < 60 mL/min/1.73 m².

### 2.3 Anthropometric measurements

Anthropometric measurements were performed by experienced physicians. Blood pressure was measured using an Omron electronic sphygmomanometer. Systolic blood pressure (SBP) and diastolic blood pressure (DBP) were recorded while the patient was seated, with the arm supported at heart level. Measurements were obtained from the non-dominant arm after a 5-minute rest. Three measurements were performed at one-minute intervals, and their average was used for analysis. Height and weight were measured indoors while the patient wore light clothing and no shoes and were recorded to the nearest 0.1 cm and 0.1 kg, respectively. Body mass index (BMI) was calculated as weight in kilograms divided by height in meters squared (kg/m²). Waist circumference (WC) was measured at the midpoint between the inferior costal margin and superior border of the iliac crest on the mid-axillary line. Calf circumference (CC) was measured with the patient standing relaxed, and the maximum circumference of the right calf was recorded to the nearest 0.1 cm.

### 2.4 Handgrip strength measurement

Handgrip strength was measured with the shoulder adducted, the upper arm against the chest, the forearm in a neutral position, and the elbow flexed at 90°. The dynamometer handle was adjusted to ensure that its base rested against the first metacarpal (heel of the palm) and the handle crossed the middle of the four fingers. Maximum grip strength of the dominant hand was assessed using an electronic grip strength dynamometer (model WCS-100, China). Three consecutive measurements were obtained at one-minute intervals, and the highest value was recorded as the final result.

### 2.5 Laboratory measurements

All patients fasted overnight before blood sample collection. Serum creatinine, cystatin C, fasting blood glucose (FBG), triglycerides (TG), total cholesterol (TC), low-density lipoprotein cholesterol (LDL-C), high-density lipoprotein cholesterol (HDL-C), and albumin levels were measured using turbidimetric immunoassay (Hitachi, Tokyo, Japan). Hemoglobin levels were assessed using the standard methemoglobin method. Serum creatinine was measured by an enzymatic method with an automatic biochemical analyzer (7600-020, Hitachi, Tokyo, Japan). Glycated hemoglobin A1c (HbA1c) was determined by high-performance liquid chromatography using a Bio-Rad D-10 system (Bio-Rad Laboratories, Hercules, CA, USA). SI was calculated as serum creatinine (mg/dL)/cystatin C (mg/L) × 100.

### 2.6 Sarcopenia risk assessment

The 2019 and 2025 Asian Working Group for Sarcopenia (AWGS) algorithm define sarcopenia using low muscle strength, low muscle quantity or quality, and low physical performance (14). Low muscle strength alone indicates probable sarcopenia, whereas confirmed sarcopenia requires low muscle quantity or quality in addition to low muscle strength. Severe sarcopenia is diagnosed when all three criteria are present. Because physical performance tests were not performed in this study, sarcopenia was defined by low muscle strength and low muscle quantity. Sarcopenia risk was defined using the AWGS 2019-recommended cut-offs, handgrip strength < 28 kg in men and < 18 kg in women, and calf circumference < 34 cm in men and < 33 cm in women.

### 2.7 Nutritional risk assessment

Nutritional risk was assessed by a dietitian using the NRS2002 tool. A score ≥ 3 indicated nutritional risk, whereas a score < 3 indicated no nutritional risk.

### 2.8 Follow-up for mortality

This retrospective study included 296 patients diagnosed with T2DM who were hospitalized at the Department of Geriatrics, Shanghai Sixth People’s Hospital from July 2019 to April 2020. Mortality data were retrieved from electronic medical records and death registries through July 30, 2020 (median follow-up: 212 days). All deaths occurring between the study enrollment date and the follow-up cut-off date were included. No patients were lost to follow-up because all had accessible medical records at the designated hospital.

### 2.9 Statistical analysis

Database management and statistical analyses were performed using SAS version 9.4 (SAS Institute, Cary, NC, USA). Continuous variables are presented as mean ± standard deviation (SD), and categorical variables are presented as counts and percentages. Between-group differences in continuous variables were assessed using the t-test, and categorical variables were compared using the chi-square test. When significant differences were detected among groups, Bonferroni-adjusted z-test was applied for *post hoc* pairwise comparisons. Correlations between the SI and other variables were evaluated using Pearson or Spearman correlation analyses, as appropriate. Analysis of variance (ANOVA) was used to compare continuous variables across multiple groups. Univariate and multivariate logistic regression analyses were used to assess the association between the SI and sarcopenia risk. Univariate and multivariate Cox proportional hazards models were used to evaluate the predictive value of the SI for mortality risk, and survival curves were generated. Both Cox proportional hazards and multivariate logistic regression models were adjusted for age, sex, and other potential confounders.

## 3 Results

### 3.1 Demographic and clinical characteristics

**Table 1** summarizes the demographic and clinical characteristics of patients with and without sarcopenia risk. The prevalence of sarcopenia risk in the study population was 78.7% (233/296). Compared with patients without sarcopenia risk, those with sarcopenia risk had significantly lower albumin, hemoglobin, BMI, WC, CC, and handgrip strength, as well as lower rates of overweight and central obesity (*P* < 0.05). In contrast, age, nutritional risk, and one-year mortality were significantly higher in the sarcopenia risk group. Sex, smoking, alcohol consumption, systolic blood pressure, diastolic blood pressure, triglyceride-glucose index (TyG), HbA1c, fasting blood glucose, total cholesterol, triglycerides, LDL-C, HDL-C, eGFR, and the prevalence of coronary heart disease, cerebral infarction, hypertension, chronic obstructive pulmonary disease, and renal dysfunction did not differ significantly between groups (*P* > 0.05).

**Table 1:** Characteristics of sarcopenia risk and N-sarcopenia risk T2DM adults.

Variables	Sarcopenia risk(n = 233)	N-sarcopenia risk(n = 63)	*P*
Age (years)	88.0 ± 5.0	84.9 ± 5.8	< 0.001
Male n (%)	180 (77.2)	48 (76.2)	0.859
Smoking n (%)	46 (19.8)	8(12.9)	0.212
Drinking n (%)	7(3.0)	2(3.2)	0.933
SBP (mm Hg)	131.4 ± 18.4	135.4 ± 21.5	0.136
DBP (mm Hg)	70.6 ± 12.2	72.1 ± 11.3	0.369
TyG	8.7 ± 0.5	8.6 ± 0.6	0.268
HbA1c (%)	6.6 ± 1.1	6.8 ± 1.2	0.218
FPG (mmol/l)	6.7 ± 2.3	6.6 ± 1.4	0.678
TC (mmol/L)	4.0 ± 1.1	4.1 ± 1.0	0.411
TG (mmol/L)	1.3 ± 0.8	1.3 ± 0.7	0.598
LDL-c (mmol/l)	2.3 ± 0.8	2.4 ± 0.9	0.434
HDL-c (mmol/l)	1.1 ± 0.3	1.1 ± 0.3	0.910
Albumin (g/dl)	37.6 ± 5.0	40.0 ± 3.7	< 0.001
Hemoglobin (g/dL)	111.7 ± 20.8	120.7 ± 22.5	0.004
eGFR(ml/min/1.73m^2^)	78.9 ± 34.5	71.4 ± 24.2	0.120
BMI (kg/m^2)^	22.9 ± 2.9	26.1 ± 3.8	< 0.001
WC (cm)	88.3 ± 9.2	96.6 ± 11.7	< 0.001
CC (cm)	27.3 ± 3.4	34.2 ± 2.6	< 0.001
HGS (kg)	10.4 ± 9.0	23.6 ± 8.3	< 0.001
Overweight n (%)	56(24.0)	44(69.8)	< 0.001
Central obesity n (%)	95(40.8)	45(71.4)	< 0.001
Coronary heart disease n (%)	177 (76.0)	47 (74.6)	0.823
Cerebral infarction n (%)	139 (59.7)	41 (65.1)	0.479
Hypertension n (%) COPD n (%)	219 (94.0) 76 (32.9)	58 (92.1) 23 (35.5)	0.580 0.592
RD n (%)	74(31.8)	20(31.7)	0.998
Nutritional risk n (%)	100(42.9)	12(19.0)	< 0.001
Mortality n (%)	58(24.9)	5(7.9)	< 0.001

SBP, systolic blood pressure; DBP, diastolic blood pressure; TyG, triglyceride-glucose index; HbA1c, Hemoglobin A1c; FPG, fasting plasma glucose; TC, total cholesterol; TG, triglyceride; LDL-c, low-density lipoprotein cholesterol; HDL-c, high-density lipoprotein cholesterol; eGFR, estimated glomerular filtration; BMI, body mass index; WC, waist circumference; CC, calf circumference; HGS, hand grip strength; COPD, chronic obstructive pulmonary disease; RD, renal dysfunction.

Data were means ± S.D. for skewed variables, or proportions for categorical variables.

### 3.2 Difference in SI between sarcopenia risk and non-sarcopenia risk groups

**Figure 1:**
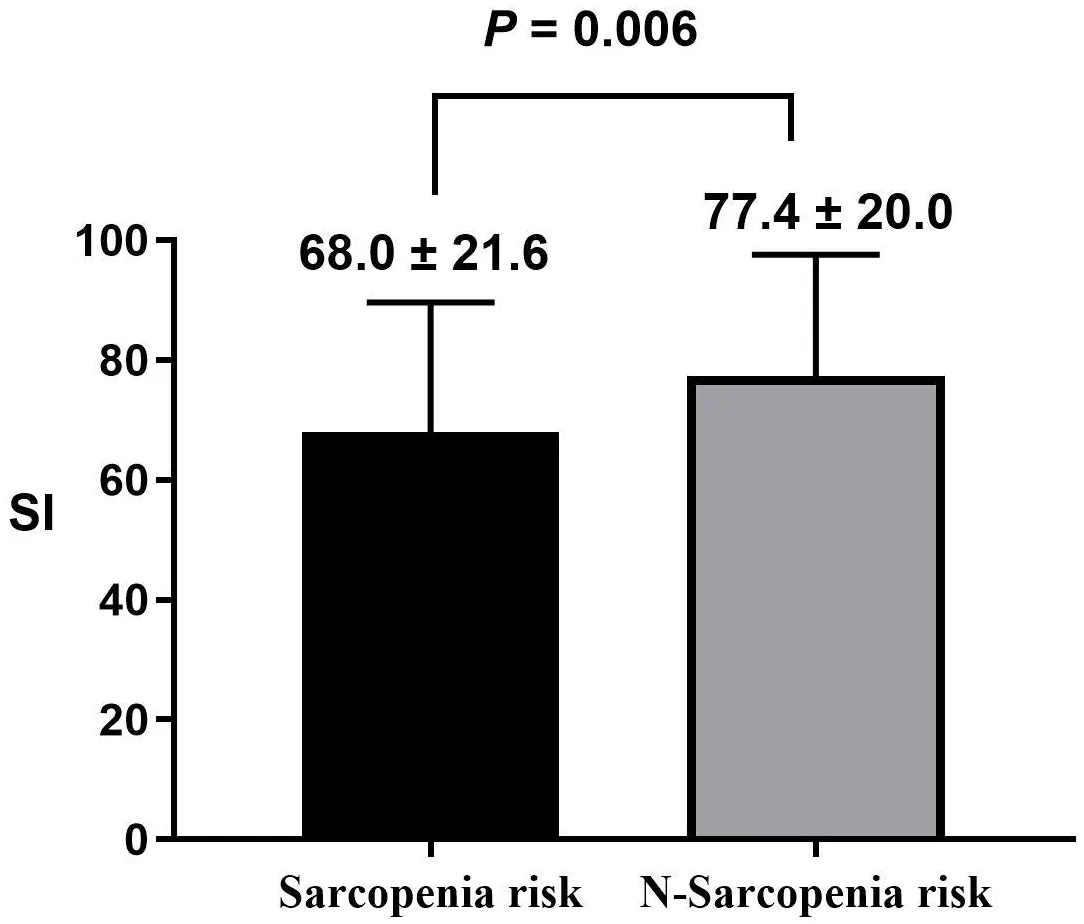
Difference of SI between sarcopenia risk and non-sarcopenia risk in T2DM.

The SI differed significantly between the sarcopenia risk and non-sarcopenia risk groups (*P* = 0.006). Among older patients with diabetes, SI was lower in the sarcopenia risk group than in the non-sarcopenia risk group (68.0 ± 21.6 vs. 77.4 ± 20.0) (**Figure 1**).

### 3.3 Association of SI with sarcopenia risk

In the unadjusted model, each SD increase in SI was associated with a 37% lower odds of developing sarcopenia risk(OR per 1-SD = 0.63, 95% CI: 0.45–0.89). After adjusting for age, sex, smoking status, and alcohol consumption status (Model 1), the SI remained negatively associated with the risk of sarcopenia risk(OR per 1-SD = 0.55, 95% CI: 0.35–0.85). Upon additional adjustment for blood lipids, overweight, central obesity, hypertension, coronary heart disease, chronic obstructive pulmonary disease, and cerebral infarction (Model 2), each SD increase in SI remained associated with a reduced risk of sarcopenia (OR per 1-SD = 0.38, 95% CI: 0.19–0.75). Finally, after further adjustment for nutritional risk and renal dysfunction (Model 3), the association persisted (OR per 1-SD = 0.32, 95% CI: 0.15–0.69) (**Table 2**).

**Table 2:** Association between SI and sarcopenia risk according to logistic regression models adjusted for potential confounders.

SI per 1-SD	Unadjusted	Model 1	Model 2	Model 3
OR (95%CI)	0.63(0.45-0.89)	0.55(0.35-0.85)	0.38(0.19-0.75)	0.32(0.15-0.69)

Model 1: Adjusted for age, sex, smoking, and alcohol consumption status.

Model 2: Further adjusted for blood lipids, overweight, central obesity, hypertension, coronary atherosclerotic heart disease, chronic obstructive pulmonary disease, and cerebral infarction based on Model 1.

Model 3: Additionally adjusted for nutritional risk and renal dysfunction based on Model 2.

### 3.4 Association of SI with all-cause mortality during follow-up

Higher SI was independently associated with a lower risk of all-cause mortality during follow-up (median 212 days). In the unadjusted model, each SD increase in SI was associated with a reduced mortality risk (HR=0.54, 95% CI: 0.41–0.70). After adjusting for age, sex, smoking status, and alcohol consumption status (Model 1), the association remained significant (HR= 0.64, 95% CI: 0.48–0.87). Further adjustment for blood lipid levels, overweight, central obesity, hypertension, coronary atherosclerotic heart disease, chronic obstructive pulmonary disease, and cerebral infarction (Model 2) showed that each SD increase in SI continued to be associated with a lower mortality risk (HR=0.67, 95% CI: 0.49–0.91). Finally, after additional adjustment for sarcopenia risk, nutritional risk, and renal dysfunction (Model 3), each SD increase in the SI remained associated with a reduced mortality risk (HR= 0.70, 95% CI: 0.48–0.97) (**Table 3**).

**Table 3:** Association between SI and mortality during the follow-up according to Cox regression models adjusted for potential confounders.

SI per 1-SD	Unadjusted	Model 1	Model 2	Model 3
SI per 1-SD HR (95% CI)	0.54(0.41-0.70)	0.64(0.48-0.87)	0.67(0.49-0.91)	0.70(0.48-0.97)

Model 1: Adjusted for age, sex, smoking, and alcohol consumption status.

Model 2: Further adjusted for blood lipids, overweight, central obesity, hypertension, coronary atherosclerotic heart disease, chronic obstructive pulmonary disease, and cerebral infarction based on Model 1.

Model 3: Additionally adjusted for sarcopenia risk, nutritional risk and renal dysfunction based on Model 2.

**Figure 2:**
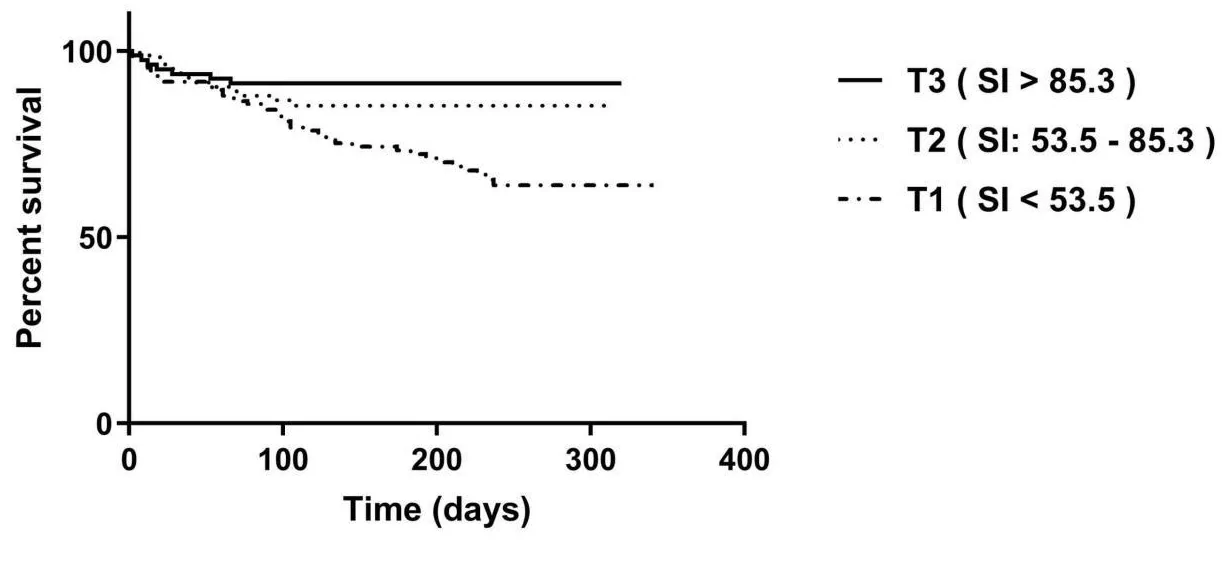
Survival curves of the study population according to the quartiles of SI. Log rank test χ2 = 15.1, P < 0.001.

**Figure 2** shows the survival curves of the participants in the different quartile groups according to the SI during follow-up (median 212 days). No significant differences were observed in the survival rates among the three groups of patients as the SI decreased, with progressive deterioration in survival outcomes. The log rank test yielded χ2 = 15.1, *P* < 0.001.

### 3.5 Cut-off value of the SI for sarcopenia risk

**Figure 3:**
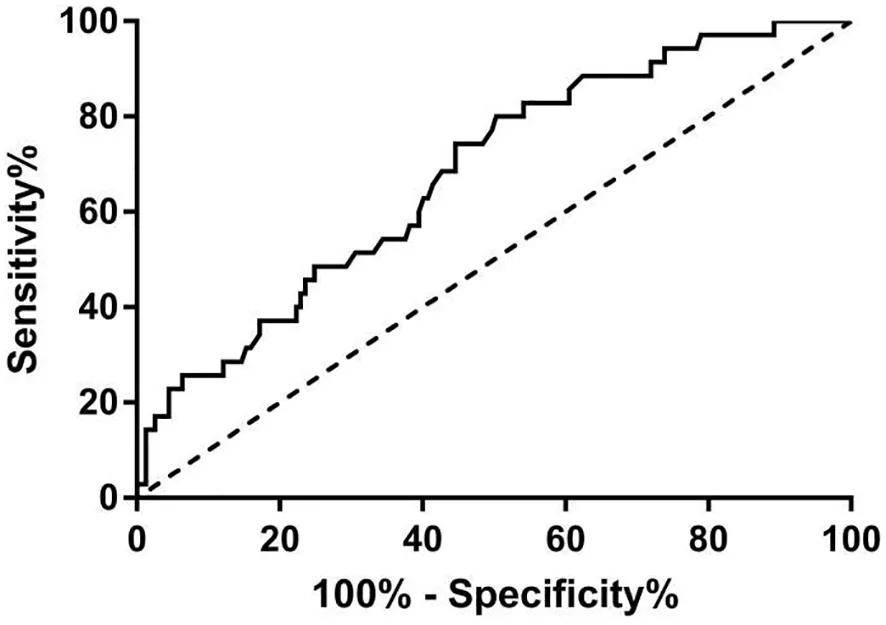
ROC analysis of SI for sarcopenia risk AUC = 0.68 (*P* < 0.001); 95% CI, 0.59–0.77; SI cut-off point = 77.3; Youden index = 0.30; sensitivity, 74.0%; specificity, 55.0%.

Receiver operating characteristic (ROC) analysis was used to determine the optimal SI cutoff for identifying sarcopenia risk in older patients with diabetes. The optimal SI cutoff was 77.3, with an AUC of 0.68 (95% CI, 0.59–0.77). Thus, in this population, an SI < 77.3 suggested sarcopenia risk(*P* < 0.001) (**Figure 3**).

Subgroup analysis stratified by renal function revealed a marked heterogeneity in the diagnostic performance of SI (**Supplementary Figure 2**). In participants with preserved renal function (eGFR ≥ 60 mL/min/1.73 m²), the optimal SI cutoff for identifying sarcopenia risk was 67.5 (AUC = 0.66; 95% CI: 0.58–0.77; *P* = 0.002), with a sensitivity of 73.0% and specificity of 58.9%. In contrast, in those with renal dysfunction (eGFR < 60 mL/min/1.73 m²), the association was attenuated (AUC = 0.54; 95% CI: 0.33–0.76; *P* = 0.634). Notably, the optimal cutoff derived from the overall cohort was substantially higher than that of the renal-normal subgroup, suggesting that creatinine retention in participants with reduced eGFR artificially inflates SI values.

## 4 Discussion

Against the backdrop of population aging, the prevalence of DM increases progressively. More than 29% of older adults aged ≥65 years have diabetes (15). Older individuals with T2DM face substantial challenges related to comorbidities, frailty, and sarcopenia, which complicate diabetes management in this age group. Moreover, sarcopenia and T2DM exhibit a bidirectional relationship, in which each condition exacerbates the other, ultimately leading to functional decline and disability (16). Age-related sarcopenia contributes to insulin resistance and the development of T2DM; conversely, patients with T2DM experience accelerated losses of muscle strength and mass compared with their non-diabetic counterparts (17).

Multiple nutritional, endocrine, inflammatory, and neurological pathways may explain how T2DM accelerates age-related declines in muscle mass and function (18). Collectively, these pathways can interfere with downstream insulin receptor signaling, impairing insulin normal actions in muscle tissue, disrupting protein synthesis, and increasing protein catabolism (19). In addition, chronic poor glycemic control, sustained oxidative stress, and complications, particularly neuropathy, can further compromise muscle energetics and exacerbate deterioration in muscle quantity and function, thereby increasing frailty risk (20). Clinically, patients with diabetes often develop skeletal muscle atrophy related to peripheral neuropathy and vasculopathy, especially in lower-limb muscle strength and function. Sarcopenia is therefore recognized as a novel complication of T2DM.

In this study, sarcopenia risk prevalence among hospitalized older Chinese patients with T2DM reached 78.7%, whereas previously reported prevalence of sarcopenia ranged from 18% to 31.1% (4, 21). This difference may reflect the advanced age of our cohort, whose mean age was approximately 85 years, and its high burden of comorbidities. This population warrants greater research attention as population aging accelerates. According to the AWGS 2019 consensus, confirmed sarcopenia requires evidence of low muscle mass as measured by DXA or BIA-derived appendicular skeletal muscle mass index, whereas low handgrip strength and low calf circumference are recommended primarily as screening tools for case finding. In the present study, we operationally defined sarcopenia risk as the combination of low handgrip strength (male <28 kg, female <18 kg) and low calf circumference (male <34 cm, female <33 cm), without direct measurement of muscle mass. Therefore, our definition does not strictly meet the AWGS 2019 diagnostic criteria for confirmed sarcopenia, and the observed prevalence (78.7%) may represent sarcopenia risk rather than disease-level certainty. Nevertheless, calf circumference has been shown to correlate significantly with appendicular skeletal muscle mass, and it is widely used as a proxy in hospital and community settings where DXA/BIA is unavailable or impractical—particularly in very old, frail, or bedridden patients. Furthermore, the recent AWGS 2025 consensus (14) has emphasized the need for harmonized diagnostic approaches across Asia and acknowledged the continued reliance on surrogate measures in clinical practice and resource-limited settings. In this context, our screening-based definition provides relevant evidence for sarcopenia risk in this particularly vulnerable population, but the results should be interpreted with caution. In our hospitalized older patients with T2DM, the prevalence is indeed higher than that in community populations, but it aligns closely with previous studies in Asian institutional and subacute care settings, which reported similarly elevated prevalence rates (22). The high burden is biologically plausible: these patients often have malnutrition, chronic inflammation, reduced mobility, and multiple comorbidities—all established risk factors for sarcopenia. Although we did not perform a sensitivity analysis, the consistency of our prevalence with that of comparable hospitalized/institutionalized cohorts suggests that the impact of this diagnostic strategy may be limited. Nevertheless, future studies using DXA or BIA and standardized AWGS criteria are essential to confirm these findings, to validate the diagnostic and identified value of the proposed SI cutoffs, and to further explore potential misclassification bias.

We found that the SI was closely associated with sarcopenia risk: lower SI values corresponded to a progressively higher risk of sarcopenia. The optimal cut-off value for sarcopenia risk screening in older patients with diabetes was 77.3, suggesting that patients with SI values below this threshold may require further diagnostic assessment using BIA, DEXA, CT, or MRI. Previous studies have demonstrated that SI is positively correlated with whole-body lean mass, appendicular lean mass, grip strength, short physical performance battery scores, and usual gait speed (23). Accordingly, the SI is considered a promising marker for predicting sarcopenia (24, 24). However, evidence regarding the SI in older patients with diabetes remains limited. Osaka et al. reported in a cross-sectional study that the creatinine-to-cystatin C ratio may serve as a risk marker for sarcopenia in Japanese individuals with diabetes, suggesting its utility as a simple screening tool for identifying patients at risk of sarcopenia (25). Another cross-sectional study suggested that the SI is associated with macro- and microvascular complications in patients with diabetes; specifically, lower SI values were linked to a greater risk of macro- and microvascular damage in the T2DM population, potentially through sarcopenia-related mechanisms (26). To our knowledge, this study is the first to demonstrate that the serum creatinine-to-cystatin C ratio-based SI identified sarcopenia risk in older Chinese patients with T2DM. Moreover, the SI was inversely associated with all-cause mortality in this population.

Several limitations of this study should be acknowledged. First, the cross-sectional design precludes causal inference regarding the relationship between sarcopenia risk and the SI. Second, we did not use DEXA, the gold-standard method, to estimate skeletal muscle mass and instead relied on CC measurements. Nevertheless, the revised consensus of the Asian Working Group for Sarcopenia recognizes CC as a surrogate marker of muscle mass (27). Third, muscle strength was assessed using grip strength. Because our participants were very old and had a high prevalence of comorbidities, gait speed and physical performance tests were not performed. Low grip strength has nonetheless been associated with activity limitations (28). However, patients with slow gait speed but normal grip strength may have been missed as sarcopenia cases in this study. Fourth, we did not collect or adjust for antidiabetic medication use. This is an important caveat, since glucose-lowering agents—especially SGLT2 inhibitors, GLP-1 receptor agonists, and insulin—may differentially influence body composition, skeletal muscle mass, renal function, and prognosis. For example, SGLT2 inhibitors can reduce body weight and possibly lean mass via caloric loss, GLP-1 receptor agonists have been associated with loss of lean body mass, while insulin may exert anabolic effects on weight and muscle. Because the present study involved hospitalized older adults with type 2 diabetes, a substantial proportion of participants were likely receiving one or more of these therapies. However, reliable medication data were not available in this dataset, and we could not evaluate their potential confounding effects. The absence of this information may introduce residual confounding, which could, in theory, either attenuate or strengthen the observed associations between sarcopenia risk and the study outcomes. Therefore, our findings should be interpreted with caution. Finally, the modest sample size and relatively small number of female participants prevented sex-stratified analyses and the determination of sex-specific identified cut-off values. Given the strong relationship between creatinine levels and muscle mass, future studies should evaluate sex-specific cutoff values and identified performance and address these limitations by expanding the sample size, using DEXA to assess skeletal muscle mass, and incorporating comprehensive medication documentation, including drug type, dose, and duration of use. The discrepancy between the overall SI cutoff of 77.3 and the renal-normal subgroup cutoff of 67.5 underscores the profound impact of kidney function on creatinine-based biomarkers. Because serum creatinine accumulates as eGFR declines, SI is inherently biased upward in individuals with renal dysfunction. Consequently, the overall cutoff may not be generalizable across all eGFR strata. Given that a lower SI value denotes sarcopenia risk, applying the higher overall threshold to a renal-healthy population would increase the likelihood of misclassifying individuals with relatively preserved muscle mass as sarcopenic, thereby potentially overestimating sarcopenia risk prevalence. Conversely, extrapolating the lower renal-normal cutoff to a mixed cohort or renal dysfunction population may underestimate sarcopenia risk, because creatinine retention raises SI values and may mask true muscle deficits, allowing affected patients to escape the diagnostic threshold. Therefore, we strongly advocate for renal-specific validation of SI prior to its clinical implementation. The generalizability of our findings also warrants careful consideration. Our study cohort comprised very old hospitalized geriatric patients with T2DM (mean age approximately 85 years), a population characterized by high comorbidity burden, malnutrition, physical frailty, and frequent renal impairment. This clinical profile differs markedly from that of community-dwelling or outpatient older adults with T2DM, who generally have fewer comorbidities, better renal function, lower sarcopenia risk burden, and a different distribution of SI values. Consequently, the proposed SI cutoffs (77.3 overall and 67.46 in the renal-normal subgroup) and their diagnostic performance may not be directly transferable to less frail, younger, or non-hospitalized populations. While our findings are clinically relevant for the identification of sarcopenia risk in this particularly vulnerable inpatient group—an area where data remain scarce—they should be applied to other care settings with caution. Indeed, the optimal SI threshold may vary according to age, disease severity, nutritional status, and kidney function. Therefore, we advocate for external validation in community and outpatient cohorts, and for future studies to determine whether setting-specific, age-specific, or renal function-stratified SI cutoffs are needed to optimize sarcopenia risk screening across the full spectrum of older adults with T2DM. Overall, this study suggests that the serum creatinine-to-cystatin C ratio-derived SI may identify sarcopenia risk in older Chinese patients with T2DM, with an optimal cut-off value of 77.3. These findings support the potential applicability of the SI for sarcopenia risk screening in older Chinese adults with T2DM.

## 5 Conclusion

SI based on the serum creatinine-to-cystatin C ratio was independently associated with both sarcopenia risk and mortality in very old Chinese inpatients with T2DM. The optimal cutoff value was 77.3. As a simple and readily available biomarker, SI may facilitate sarcopenia risk screening in this high-risk population and serve as a convenient tool for monitoring treatment responses in clinical practice.

## Data Availability

The raw data supporting the conclusions of this article will be made available by the authors, without undue reservation.
