# Supplementary material for: Sarcopenia index (serum creatinine/cystatin C ratio) independently identifies sarcopenia risk and mortality in older hospitalized patients with type 2 diabetes mellitus: a retrospective analysis
Source: Front Endocrinol (Lausanne). 2026 Sep 1;17:1940459. doi: 10.3389/fendo.2026.1940459 (PMC13574638; doi:10.3389/fendo.2026.1940459)
Supplement: Supplementary file 1 [file SupplementaryFile1.doc]

T2DM patients visiting the Geriatrics Department (n=425)

Exclusion criteria: patients aged <65 years (n=2), those undergoing hemodialysis (n=3), peritoneal dialysis (n=1), patients with acute renal insufficiency (n=3), patients with a history of malignant tumor or cachexia (n=15), and patients in critical condition (n=69), including those with acute myocardial infarction, acute heart failure, acute stroke, or severe infection; patients unable to communicate (n=19); and bedridden patients (n=17) with edema.

Inclusion criteria:
Age ≥65 years; complete nutritional risk and sarcopenia assessment available

Final cohort of enrolled patients (n=296)

Follow-up to July 30, 2020

Number of follow-up participants (n=296); no loss to follow-up occurred.

Supplementary Figure 1. Flowchart of study process

Patients were excluded based on the following criteria: (1) age <65 years (n = 2); (2) renal replacement therapy, including hemodialysis (n = 3) and peritoneal dialysis (n = 1); (3) critical illness (n = 69), defined as acute myocardial infarction, acute heart failure, acute stroke, or severe infection; (4) a history of malignant tumor or cachexia (n = 15); (5) inability to communicate (n = 19); and (6) bedridden status with edema (n = 17). Ultimately, 296 older inpatients with T2DM were enrolled.

**Supplementary** **F**ig**ure 2A**

Supplementary Figure 2A. ROC analysis of SI for sarcopenia in participants with non-renal dysfunction AUC = 0.68 (*P* = 0.002); 95% CI, 0.58–0.77; SI cut-off point = 67.5; Youden index = 0.32; sensitivity, 73.0%; specificity, 58.9%.

**Supplementary Figure 2B**

Supplementary Figure 2B. ROC analysis of SI for sarcopenia in participants with renal dysfunction AUC = 0.54 (*P* = 0.634); 95% CI, 0.33–0.76, indicating a non-significant discriminative ability.
